# High throughput generation of promoter reporter (GFP) transgenic lines of low expressing genes in Arabidopsis and analysis of their expression patterns

**DOI:** 10.1186/1746-4811-6-18

**Published:** 2010-08-06

**Authors:** Yong-Li Xiao, Julia C Redman, Erin L Monaghan, Jun Zhuang, Beverly A Underwood, William A Moskal, Wei Wang, Hank C Wu, Christopher D Town

**Affiliations:** 1 J. Craig Venter Institute, 9704 Medical Center Drive, Rockville, MD 20850, USA

## Abstract

**Background:**

Although the complete genome sequence and annotation of Arabidopsis were released at the end of year 2000, it is still a great challenge to understand the function of each gene in the Arabidopsis genome. One way to understand the function of genes on a genome-wide scale is expression profiling by microarrays. However, the expression level of many genes in Arabidopsis genome cannot be detected by microarray experiments. In addition, there are many more novel genes that have been discovered by experiments or predicted by new gene prediction programs. Another way to understand the function of individual genes is to investigate their *in vivo *expression patterns by reporter constructs in transgenic plants which can provide basic information on the patterns of gene expression.

**Results:**

A high throughput pipeline was developed to generate promoter-reporter (GFP) transgenic lines for Arabidopsis genes expressed at very low levels and to examine their expression patterns *in vivo*. The promoter region from a total of 627 non- or low-expressed genes in Arabidopsis based on Arabidopsis annotation release 5 were amplified and cloned into a Gateway vector. A total of 353 promoter-reporter (GFP) constructs were successfully transferred into Agrobacterium (GV3101) by triparental mating and subsequently used for Arabidopsis transformation. Kanamycin-resistant transgenic lines were obtained from 266 constructs and among them positive GFP expression was detected from 150 constructs. Of these 150 constructs, multiple transgenic lines exhibiting consistent expression patterns were obtained for 112 constructs. A total 81 different regions of expression were discovered during our screening of positive transgenic plants and assigned Plant Ontology (PO) codes.

**Conclusions:**

Many of the genes tested for which expression data were lacking previously are indeed expressed in Arabidopsis during the developmental stages screened. More importantly, our study provides plant researchers with another resource of gene expression information in Arabidopsis. The results of this study are captured in a MySQL database and can be searched at http://www.jcvi.org/arabidopsis/qpcr/index.shtml. Transgenic seeds and constructs are also available for the research community.

## Background

At the end of 2000, the first plant genome project, sequencing of the whole genome of *Arabidopsis thaliana*, was completed by a multinational collaborative effort [1]. Subsequently, the plant scientific community set the goal of understanding the function of each gene in the Arabidopsis genome, which is encapsulated in the National Science Foundation (NSF) 2010 program, and this challenge has been taken up by many Arabidopsis researchers [2]. Identification of each gene in the fully sequenced Arabidopsis genome and uncovering their function will provide crucial information for biologists to understand plant physiology, genetics, development and evolution. One way to understand the function of genes on a genome-wide scale is expression profiling by microarrays using formats that include complementary DNA [3], oligonucleotides [4,5] or amplicons [6]. Recently, whole genome tiling arrays have been developed and used to interrogate the gene structure and expression status of the entire Arabidopsis genome [7-9]. In addition, microarrays have been used to analyze genome features such as chromatin structure [10,11]; sites of DNA modifications [12,13]; and DNA-protein binding sites [14,15]. So far, the expression data from ATH1 chips covers a wide range of experimental treatments and conditions in the public domain http://affymetrix.arabidopsis.info/narrays/experimentbrowse.pl; http://arabidopsis.org/info/expression/ATGenExpress.jsp that collectively make a great contribution to understanding gene function in Arabidopsis. Another way to understand the function of individual genes is to investigate their *in vivo *expression patterns spatially, temporally, or conditionally by reporter construct methods that include 1) random integration of promoter-less reporter constructs into the genome and monitoring of their expression to identify genes, promoters, and enhancers [16]; 2) transformation of promoter-reporter fusions from a particular gene back into the organism and observing the reporter expression patterns [17-22].

Many studies on gene expression in plants have shown that the majority of elements necessary for and important for the regulation of expression lie immediately upstream of the transcriptional initiation site, usually within the first kilobase (17-21). Although there are reports showing that the gene regulatory signals may also be located in other regions (e.g. within introns or the 3' UTR) [23-25], these are in a minority. Thus the traditional view that the majority of a plant promoter's activity as being immediately upstream of the transcriptional initiation site is generally well supported and there is considerable evidence that the *in vivo *expression of reporter genes driven by such regions (1-2 kb upstream) does reflect the expression pattern of the native gene. It can thus provide preliminary but critical information on endogenous gene expression patterns and evidence of their biological or developmental functions. A transgenic system such as this can also reveal cell-type-specific patterns of gene expression (e.g. in trichomes, hydathodes or stomata) without a priori knowledge which would escape detection by microarray approaches.

In our current study, we focused on two groups of genes in Arabidopsis, 1) unannotated genes identified by our previous experiments [26] that were predicted by EuGene [27] and Twinscan [28], programs that incorporate comparative genomics information. 2) Annotated Arabidopsis genes whose expression was detected in less than 5% of ~1,400 ATH1 Affymetrix GeneChip experiments downloaded from The Arabidopsis Information Resource (TAIR) website at the start of this work. For both groups of genes, our knowledge of their function is extremely limited. In our previous study that focused on hypothetical gene structure in Arabidopsis, localized expression patterns of five exemplar genes were detected by cloning the promoter regions into green fluorescent protein (GFP) reporter constructs and transforming them into Arabidopsis [29]. For the present study, we developed a high throughput pipeline to create promoter-reporter (GFP) fusions of both gene groups, transformed them into Arabidopsis, and screened the expression of the reporter genes in positive transgenic plants at various developmental stages. Over 600 Arabidopsis genes were selected to make the promoter-reporter constructs and transgenic lines were obtained for just under half of these. The expression of promoter-reporter (GFP) constructs in transgenic plants was examined at 4 developmental stages: on the selection plate around 10 days after germination, at the rosette stage in soil, just before flowering, and at the flowering stage. GFP expression has been observed in constructs from 150 different promoters and is typically localized to a few tissues or cell types (e.g. hydathode, pedicel, socket cell, guard cell), consistent with the absence from or low abundance of transcripts from these genes in EST libraries. For the remaining target genes, no visible expression was detected, although PCR confirmed the presence of the transgene in all cases tested. All the expression patterns have been annotated according to plant ontology codes http://www.plantontology.org/ and stored in a MySQL database. To our knowledge, this is the first set of large-scale promoter-reporter expression data in Arabidopsis focusing on novel genes and genes with limited expression data, and thus should be a valuable resource for the plant research community. All our data are publicly available through the project website http://www.jcvi.org/arabidopsis/qpcr/index.shtml; transgenic seeds, and constructs are also available for research community through the Arabidopsis Biological Resource Center (ABRC).

## Results

### Selection of target genes for promoter-reporter analysis

The selection of target genes was based on the Arabidopsis annotation release 5 that was available when the project started and includes 1) previously identified un-annotated genes; 2) genes represented on the Affymetrix ATH1 GeneChip showing no expression; 3) genes represented on the Affymetrix ATH1 GeneChip showing very limited expression. Un-annotated genes are genes in the intergenic regions whose expression was detected by RACE in our previous experiments and those predicted in intergenic regions by EuGene [27] and/or Twinscan [28]. Genes on the Affymetrix ATH1 GeneChip showing no or limited expression were identified from the Affymetrix MAS calls of 1381 ATH1 arrays downloaded from TAIR, combined with massively parallel signature sequencing (MPSS) data available at that time [30] (for details see MATERIAL AND METHODS). The resulting set of genes (identifiers and targeted promoter sequences) is presented in additional file 1: Table S1. Many of these were not annotated at the time of this work. However, all except 27 are now present in the TAIR9 annotation (June 2009) (additional file 2: Table S2). Five of these 27 still unannotated genes showed GFP expression in transgenic plants.

### Development of a high throughput pipeline

**Figure 1:**
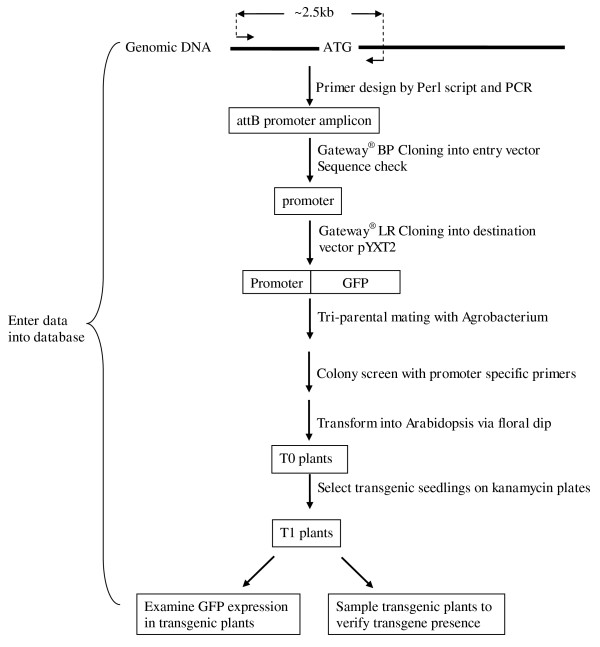
**Flow scheme for generation of promoter reporter (GFP) transgenic lines**.

**Figure 2:**
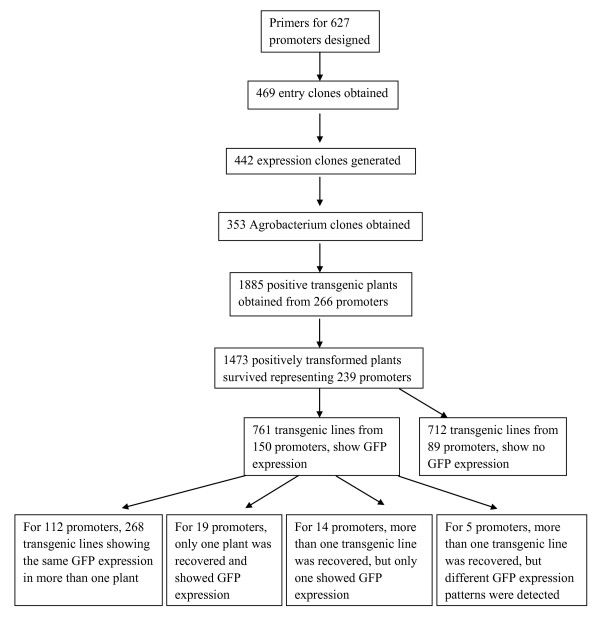
**Outcome of generation of promoter reporter (GFP) transgenic lines**. The figure shows the numbers of genes for which primers were designed, the success rate of high throughput cloning, transformation and the outcome of transgenic lines.

To handle the relatively large number of genes to be studied, we modified our original protocols [29,31] to produce a robust high throughput pipeline that included batch primer design, high throughput cloning and transformation, and a project-tracking LIMS implemented in a MySQL database (Figure 1). In this study, the Gateway^® ^Gene Cloning strategy [32] was used to make promoter-reporter constructs. PCR amplification of promoters and Gateway cloning were performed in 96-well plate format. Two colonies from each Gateway BP cloning reaction (from each promoter) were picked and sequenced to confirm target identity. DNA isolated robotically from the sequence-confirmed clones was used in Gateway LR reactions. The triparental mating method [33] was used to transfer promoter-reporter constructs from *E. coli *to Agrobacterium (GV3101). Bypassing the step of destination clone DNA isolation from *E. coli *made this step economical and large scale. In addition, we cultured only 50 ml of Agrobacterium for plant transformation and did the floral dip in 50 ml Falcon tubes. As shown in Figure 2, a total of 627 candidate genes were put through the construction and transformation pipeline. Gateway entry clones were obtained from 469 genes and sequence confirmed. Of these, 442 were successfully transferred into the destination vector, pYXT2 containing the GFP reporter gene [34]. Subsequently, Agrobacterium clones from 353 genes were obtained by tri-parental mating, and then transformed into Arabidopsis by the floral dip method [35]. Three independent transformations (floral dips with separate plants) were performed for each construct and 3 seedlings from each kanamycin selection plate (whenever possible) were transferred into soil for maximum possible set of 9 transformed plants per construct. Positive transgenic plants from 266 constructs representing promoters from 266 genes were obtained and GFP expression patterns have been observed from 150 constructs in at least one of the 4 developmental stages examined. This newly developed cloning and transformation pipeline greatly improved the throughput and lowered the effort compared with our previous work.

Additionally, we have developed a project specific MySQL database to record and track the large amount of data generated by this project, including target genes, promoter selection, primer design, different steps of clone tracking, transgenic plant tracking, and GFP image capture. All the GFP images were annotated according to Plant Ontology (PO) codes http://www.plantontology.org/index.html[36]. This MySQL database supports our publicly accessible website http://www.jcvi.org/arabidopsis/qpcr/index.shtml which users can search for GFP images by tissue type, locus name, or PO code.

### Expression patterns of promoter-reporter (GFP) constructs

**Figure 3:**
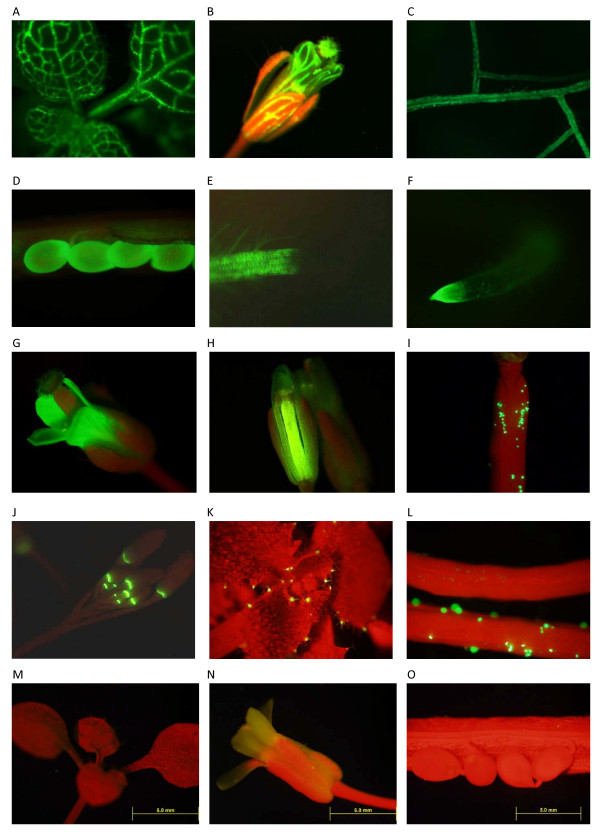
**Examples of the expression patterns from different promoter-reporter constructs**. A. leaf vascular expression from promoter-report construct of gene AT.CHR1.16.7; B. sepal and petal expression from promoter-report construct of gene AT.CHR1.16.7; C. root expression from promoter-report construct of gene NOVEL_CHR1_5915494; D. seed expression from promoter-report construct of gene NOVEL_CHR1_5915494; E. root but not root tip expression from promoter-report construct of gene AT1G64820; F. root tip expression from promoter-report construct of gene AT4G13985; G. petal expression from promoter-report construct of gene AT2G17845; H. carpel expression from promoter-report construct of gene AT2G40250; I. pollen expression from promoter-report construct of gene AT2G24370; J. expression from promoter-report construct of gene AT4G18395 in the region of the flower abscission zone; K. hydathode expression from promoter-report construct of gene AT02EUG13430; L. pollen expression from promoter-report construct of gene AT2G24370 (lower silique) comparing to wild-type pollen (upper silique); some un-florescent pollens on the low silique are the wild-type pollens spreading from the upper one. M. wild-type leaf without GFP expression; only chlorophyll autofluorescence is visible. N. wild-type flower without GFP expression; chlorophyll autofluorescence is visible. O. wild-type seeds without GFP expression; chlorophyll autofluorescence is visible.

In total, 1,885 positive transgenic lines from 266 promoter-reporter constructs were obtained. However, only 1,457 transgenic plants from 239 genes survived the process of transferring plants from selection plates to soil. Among them, 761 transgenic plants from 150 promoters showed GFP expression. Of these 150 promoters, consistent GFP expression patterns were observed in two or more transgenic plants for 112 promoter constructs. For 19 promoter constructs, only one transgenic line was recovered and showed GFP expression. For 14 promoter constructs, more than one transgenic line was obtained, but only one line expressed GFP. In addition, plants from 5 promoters had inconsistent GFP expression patterns. These results are summarized in Figure 2. Among the 112 promoter constructs that displayed the same GFP expression pattern from more than one transgenic plant, promoters from 79 genes showed the same GFP expression patterns in transgenic plants obtained from different floral dip events, providing high confidence of the location of expression of the transgene. GFP expressing lines for 27 different constructs were randomly chosen for promoter re-amplification using flanking primers and the PCR products were sequenced. All were confirmed to be from the intended promoters as shown in additional file 2: Table S2. In addition, leaf PCR [37] using GFP primers was performed on 423 transgenic lines, from 83 promoters, that did not show GFP expression. All had positive amplification indicating the presence of the GFP construct in these lines. In total, we recorded 2,287 GFP expression images and described their expression patterns with Plant Ontology (PO) codes. A total of 3,371 PO codes were assigned, as more than one PO code referencing different parts of the plant could be assigned to one image, and collectively these represented eighty-one different expression patterns (Table 1). The leaf vascular system was the most frequently annotated code and was assigned to 38 promoters. For example, the Twinscan-predicted At.chr1.16.7 promoter construct shows expression in vascular tissue from leaf (PO:0000036, Figure 3A), petal (PO: 0000054) and sepal (PO:0004723, Figure 3B). Examples of multiple PO codes/expression patterns from one construct include the novel_chr1_5915494 (a novel gene discovered in our previous project) which is expressed in root (PO:0009005, Figure 3C) and seed (PO:0009010, Figure 3D). Some expression patterns which we detected are very specific. The promoter-reporter construct from AT1G64820 is expressed throughout the root but excluding the root tip (Figure 3E), while the construct from AT4G13985 was expressed only in the root tip (Figure 3F). Many instances of GFP expression were detected in floral organs, such as petal expression from promoter-report construct of AT2G17845 (Figure 3G) and carpel expression from that of AT2G40250 (Figure 3H). A listing of all genes assigned to each PO code is provided in additional file 3: Table S3

**Table 1:** Number of expression patterns obtained categorized by PO codes.

PO_code	PO_code_name	Number of expressed promoters
PO:0000036	Leaf vascular system	38

PO:0009005	root	37

PO:0005660	hydathode	35

PO:0000013	cauline leaf	32

PO:0003011	root vascular system	32

PO:0000056	floral bud	26

PO:0006504	leaf trichome	25

PO:0009052	pedicel	24

PO:0009032	petal	22

PO:0006502	flower abscission zone	21

PO:0006325	inflorescence node	20

PO:0000282	trichome	20

PO:0009031	sepal	19

PO:0000054	petal vascular system	17

PO:0009001	fruit	14

PO:0020139	midvein	14

PO:0000293	guard cell	12

PO:0009049	inflorescence	12

PO:0009047	stem	12

PO:0006056	cotyledon epidermis	11

PO:0006016	leaf epidermis	11

PO:0000112	stem epidermis	11

PO:0000115	socket cell	10

PO:0000146	abscission zone	9

PO:0004707	fruit dehiscence zone	9

PO:0009010	seed	9

PO:0004723	sepal vascular system	9

PO:0009067	filament	8

PO:0020127	primary root	8

PO:0006040	sepal epidermis	8

PO:0000039	stem vascular system	8

PO:0000034	vascular system	8

PO:0006501	leaf abscission zone	7

PO:0020128	leaf margin	7

PO:0000332	pavement cell	7

PO:0005003	stem trichome	7

PO:0000035	cotyledon vascular system	6

PO:0005679	epidermis	6

PO:0009025	leaf	6

PO:0006036	root epidermis	6

PO:0009030	carpel	5

PO:0000025	root tip	5

PO:0009015	vascular tissue	5

PO:0020030	cotyledon	4

PO:0004536	fruit pedicel	4

PO:0009072	ovary	4

PO:0000052	petiole vascular system	4

PO:0000256	root hair	4

PO:0009046	flower	3

PO:0004724	hypocotyl-root junction	3

PO:0009081	inflorescence branch	3

PO:0005028	inflorescence vascular system	3

PO:0005645	leaf mesophyll	3

PO:0009053	peduncle	3

PO:0005021	sepal margin	3

PO:0000033	valve	3

PO:0005011	anther dehiscence zone	2

PO:0005008	fruit septum	2

PO:0008003	fruit vascular system	2

PO:0020100	hypocotyl	2

PO:0006019	leaf abaxial epidermis	2

PO:0004006	mesophyll cell	2

PO:0000051	petiole epidermis	2

PO:0020031	radicle	2

PO:0020141	stem node	2

PO:0004711	axillary inflorescence bud	1

PO:0005019	carpel vascular system	1

PO:0006338	embryonic leaf	1

PO:0008015	hypocotyl vascular system	1

PO:0006339	juvenile leaf	1

PO:0020121	lateral root	1

PO:0000017	leaf primordium	1

PO:0006034	leaflet margin	1

PO:0020091	male gametophyte	1

PO:0005012	pedicel vascular system	1

PO:0006041	petal epidermis	1

PO:0006081	primary root apical meristem	1

PO:0006085	root meristem	1

PO:0000014	rosette leaf	1

PO:0009073	stigma	1

PO:0020041	stipule	1

Note that a single line may be characterized by more than one PO code.

## Discussion

The aim of our study was to examine the *in vivo *expression of many Arabidopsis genes of unknown function that show little or no expression based on EST, MPSS, or microarray data using reporter (GFP) genes driven by their native promoter and thus learn something about their potential function. In this study, we selected 627 Arabidopsis genes with unknown function, based on TIGR5 genome annotation, including unannotated genes located in the intergenic regions revealed in our previous RACE experiments [26], intergenic genes predicted by EuGene [27] and/or Twinscan [28], and annotated genes with zero or less than 5% "present" gene calls from the 1,381 ATH1 arrays available at the start of these experiments. We developed a high throughput pipeline to generate promoter-reporter (GFP) constructs and transform them into Arabidopsis.

### Selection of expression vector

At the outset, three Gateway compatible binary vectors were compared for the expression level of their reporter genes and their convenience for this project: 1) pBGWFS7 http://gateway.psb.ugent.be/search/index/transcriptional_reporters/any which contains dual reporter genes (GFP and GUS) with BASTA selection, 2) pYXT1 which contains a GUS reporter gene with kanamycin selection, and 3) pYXT2 which contains the eGFP reporter gene [38] with kanamycin selection. We used both pYXT1 and pYXT2 vectors successfully in our previous study [29]. For comparison, we cloned 5 promoters into each of these vectors and found that, although the dual reporter vector, pBGWFS7 that contains the BASTA selection marker is simplest for selection of the transgenic lines, only GUS expression and no GFP expression was detected. For pYXT1 and pYXT2 vectors, both GUS and GFP expression were detected equally well. However, we chose to use the pYXT2 vector because it eliminates the need for GUS staining that would increase the amount of effort required in these experiments.

### High throughput procedure

A high throughput method was developed to facilitate the ease of cloning and transformation over traditional gene-by-gene methods such as that used previously [29]. It includes a Perl script to design Gateway-compatible primers for cloning the promoter regions of candidate genes, PCR amplification of promoters of candidate genes, Gateway BP cloning, and Gateway LR cloning all in 96-well format, Agrobacterium transformation by tri-parental mating and floral dipping in 50 ml Falcon tubes (see MATERIALS AND METHODS). The 5' ("left") primer is located at least 2000 bp upstream of the start position of the coding sequence of the candidate genes since many studies have shown that region 1-2 kb upstream of the translation start site of genes determines the specificity of gene expression [39-44]. The 3' ("right") primers for the promoters were all located between 50 bp and 150 bp downstream of the translation start site of the target genes allowing an in-frame fusion with the GAL4-VP16 component of the reporter construct that contained at least 16 amino acids of the studied gene's coding sequence as well as the 8 amino acid linker from Gateway system. This script made the primer design process facile, reliable and consistent.

Positive BP clones were picked and sequenced to confirm the cloned promoter sequences. Residual BP DNA from the sequence confirmation step was used for the Gateway LR cloning reaction in 96-well plate format. The triparental mating method was chosen to transfer the Gateway expression clones from *E. coli *to Agrobacterium [33,45], eliminating the need for DNA isolation from the selected clones as well as the subsequent high throughput electroporation. For the plant transformation, we cultured only 50 ml Agrobacterium, which allowed all steps (precipitation and resuspension of Agrobacterium cells and floral dipping of Arabidopsis plants) to be carried out in single 50 ml Falcon tubes. Recently, Davis et al [46] successfully transformed Arabidopsis by dipping plants directly into Agrobacterium cultures supplemented with surfactant, eliminating the need for media exchange to a buffered solution and further simplifying the transformation process.

A very important feature of this pipeline was the creation of a project specific laboratory information management system (LIMS) in a MySQL relational database to track all stages of the pipeline: candidate genes, primers, different stages of clone construction, transgenic lines, GFP checking at different stages, PCR and sequencing results and annotation of all the GFP images.

Therefore, this high throughput cloning and data tracking pipeline made our project management efficient and robust. However, out of 627 targeted promoters, only 266 constructs were ultimately transferred into Arabidopsis plants. This overall success rate is due to a degree of failure at each experimental step. For example, the successful rate for obtaining entry clones by BP cloning is 74.8%, for expression clones from entry clones by LR cloning 94.4%, for Agrobacterium clones by triparental mating 79.7%, and for kanamycin-resistant transgenic plants by floral dip 75.4%. Because of the nature of this high throughput project, we have not yet repeated any experimental step. Certainly the overall successful rate will increase if the unsuccessful clones at each step are reprocessed.

### Expression Pattern Analysis

In order to confirm that the expression patterns are from the intended cloned promoters, vector-based primers flanking the cloning site were used to amplify the cloned promoters from transgenic plants showing GFP expression and the PCR products were sequenced for confirmation. Of 27 different constructs showing GFP expression, all the promoters were verified as correct. However, since not all lines were tested, researchers may wish to perform their own confirmation before using our lines. There are a total of 112 promoter-reporter constructs that show the same expression in more than one plant. For 79 promoter-reporter constructs, the same GFP expression patterns were observed from transgenic lines derived from independent floral dips and the other 33 constructs produced the same GFP expression patterns from separate seed borne by a single dipped plant are thus the most reliable data set. It has been shown that female reproductive tissues are the primary target of Agrobacterium-mediated transformation and that the transformants derived from the same seed pod contain independent T-DNA integration events [47].

The validity of the specific patterns of GFP expression from a representative set of promoters was confirmed by quantitative real-time PCR (qRT-PCR) on RNA samples from multiple tissues (additional file 4: Table S4). In every case, the tissue showing the highest expression (lowest Ct) by qRT-PCR was the one from which GFP expression was observed, and in almost every case this expression value was many times higher than any of the other tissues examined.

In this study, we checked GFP expression at 4 different stages: on the selection plate around 10 days after germination, at the rosette stage in soil, just before flowering, and at the flowering stage. These stages were chosen both to cover several developmental stages and also for the convenience of the large amount of GFP screening and to and minimize the stress for the T0 transgenic plants (e.g. checking root GFP on the selection plates and during transplanting to soil). If the kanamycin-selected transgenic plants did not show any GFP expression at any of the stages examined, PCR with GFP specific primers was used to confirm the presence of the reporter transgene. Out of 256 plants representing 89 promoter reporter constructs without GFP expression that we tested by leaf PCR [37], all were positive with GFP primers. There are several possible reasons for the lack of detectable GFP expression in these lines. The promoter might be active only under conditions or at specific developmental stages not examined in this study. Alternatively, in contrast to the localized expression seen with many of the promoters, those without visible GFP expression may in fact be expressed in the plant but at levels too low to be detected by this method. It is also possible that some of the promoter-reporter constructs were truncated or rearranged during T-DNA integration [48], or that gene silencing occurred [49]. In addition, the inconsistent GFP expression patterns that were detected from different transgenic lines of 5 promoter constructs may be due to position effects or to truncation or re-arrangement of the constructs during transformation as well as to human error.

The goal of this project was to use the expression of promoter-reporter constructs in transgenic plants to infer the function of these no/low expression genes. Promoters from 35 genes tested had GFP expression in hydathodes, a secretory structure on leaf margins. An example of hydathode expression from promoter-report construct of gene AT02EUG13430 is shown in Figure 3K. Studies have shown that some genes expressed in hydathodes are related to plant tolerance to toxicity. For example, the Bot1 gene in barley is responsible for boron-toxicity tolerance [50], the MTP11 gene in Arabidopsis is associated with plant tolerance to manganese [21], and AtHMA3, a P_1B_-ATPase protein plays a role in the detoxification of heavy metals [51]. AtCML9, a calmodulin-like protein from *Arabidopsis thaliana*, can alter plant responses to abiotic stress and abscisic acid and the expression of its promoter-reporter construct also included hydathodes [43]. In addition, hydathodes are one of the expression locations of a promoter-reporter construct from ECA3, a Golgi-localized P2A-type ATPase that plays a crucial role in manganese nutrition in Arabidopsis [44]. Thus, it is possible that some the genes of unknown function analyzed in this study that show hydathodes expression are also involved in tolerance or detoxification pathways, suggesting a direction for further study. Motif search by Multiple Em for Motif Elicitation (MEME) [52] for all promoter sequences with hydathode expression found a motif of CTTAAGA (P = 8.67e-09). However, its function and specificity will require experimental verification.

Twenty six promoter-reporter constructs expressed GFP in the abscission zones of siliques, flowers and leaves including expression around the flower abscission zone from construct of gene AT4G18395 (Figure 3J). Abscission is a physiological process that involves the programmed separation of entire organs, such as leaves, petals, flowers, and fruit, allows plants to discard nonfunctional or infected organs, and promotes dispersal of progeny [53]. Promoter-reporter constructs from a number of confirmed abscission related genes including BOP1 [54], BFN1 [55,56], HAE, HSL2, MKK4,5 [53], AtZFP2 [57] show similar expression at abscission zones. Using MEME [52], the sequence TAACCACTCA was the most significant motif found in the promoters analyzed in this study.

Thirty-six promoter-reporter constructs are expressed in trichomes or the socket cells that surround a trichome and provide support, suggesting their possible function in trichome development, expansion and branching. Many promoter constructs in our study were expressed in specific floral organs, including sepal, petal, filament, anther, carpel, and pollen. For example, pollen specific GFP expression was detected from the construct of gene AT2G24370 (Figure 3I, L). In addition to providing the clues to their function, they may also provide novel promoters for plant genetic engineering. For example, it has been shown that completely sterile Arabidopsis plants can be generated by engineering carpel and stamen-specific expressed genes [58]. Use of the Ory s1 promoter (pollen-specific promoter) with antisense Lol p5A cDNA led to the production of hypoallergenic rye grass (*Lolium perenne*) [59].

Overall, in our study, positive transgenic plants were obtained from 266 promoter constructs derived from our intergenic and non- or low-expressing genes of unknown function. Among them, about 56% of constructs showed GFP expression in Arabidopsis. Thus the *in vivo *expression data from promoter-reporter constructs generated in this study has provided insights into possible functions of many genes previously lacking both expression data and functional annotation as well as another great gene expression resource for the research community.

## Methods

### Selection of the genes on the Affymetrix ATH1 GeneChip showing no or very limited expression

ATH1 no- or low-expression genes were identified as follows. The results of 1381 ATH1 arrays were downloaded from TAIR and the Affymetrix MAS calls used to classify them as "expressed" (present call) or "non-expressed" (marginal or absent call) in each experiment. "ATH1 no expression genes" and "ATH1 low expression genes" are those showing expression in either none or in less than 5% of the experiments respectively. Candidate genes from the microarray analysis were excluded if they were shown by massively parallel signature sequencing (MPSS) [30] to be expressed.

### Primer design

The Perl script for primer design for promoter cloning has following features 1) align the CDS of the candidate genes to the Arabidopsis genomic sequence to locate the ATG start codon using BLAT; 2) extract 3000 bp upstream of the ATG and 150 bp downstream of the ATG as the genomic target region; 3) invoke Primer3 http://primer3.sourceforge.net/ to design primers using PRIMER_PRODUCT_SIZE_RANGE '2150-2650' and TARGET '3000, 50' which locates the "left" primer at least 2000 bp upstream of ATG start codon and the "right" primers at least 50 bp downstream of the start codon; 4) check the specificity of potential primer pairs against the Arabidopsis genome using BLAST [60] using 80% identity and allowing a maximum of 3 hits in the genome per pair of primers (it will hit itself in the genome). The script iterates through primer pair design until these criteria are fulfilled. In addition, the user can directly input Gateway sequences at the ends of upstream and downstream primers (aaaaagcaggct is added to the 5' end of upstream primers and agaaagctggt to the 5' end of downstream of primers). Thus, the output of this script will be the primers with gene specific sequences at 3' ends and Gateway cloning sequences at 5' ends in the format for plate ordering according to the primer manufactures' requirements. This primer design script is available request to the authors.

### Promoter-reporter (GFP) construct production

The Gateway cloning strategy was used to make promoter-reporter constructs largely according to the protocols in the Gateway Cloning Technology booklet (Invitrogen, Carlsbad, CA). Promoter amplifications were performed in 96 well plate format and in 2 PCR stages in order to add Gateway cloning sequences at the ends. The first PCR reaction contains 13.4 μL H_2_O, 4.0 μL 5× HF buffer, 0.40 μL 10 mM dNTPs, 2.0 μL gDNA (10 ng/uL) and 0.2 μL Phusion/iProof enzyme (New England Bio Lab, Ipswich, MA). The PCR conditions are: 1×: 98°C 30 sec, 1×: 98°C 10 sec, 63°C 30 sec, 72°C 2 min, 1×: 98°C 10 sec, 62°C 30 sec, 72°C 2 min, 1×: 98°C 10 sec, 61°C 30 sec, 72°C 2 min, 1×: 98°C 10 sec, 60°C 30 sec, 72°C 2 min, 1×: 98°C 10 sec, 59°C 30 sec, 72°C 2 min, 9×: 98°C 10 sec, 58°C 30 sec, 72°C 2 min, 1×: 72°C 10 min, 1×: 4°C forever. The primers used in the 1^st ^PCR reaction were from the primer design script and contain gene-specific sequence as well as the attB site sequence for BP cloning. The second PCR reaction contains: 18.8 μL H_2_O, 6 μL 5× HF buffer, 0.8 μL 10 mM dNTPs, 4.0 μL AttB Primer mix10 uM, 0.4 μL Phusion/iProof (New England Bio Lab, Ipswich, MA), and 10 μl from the PCR1 reaction was used as template. The 2^nd ^PCR conditions are: 1×: 98°C 30 sec, 19×: 98°C 10 sec; 56°C 30 sec; 72°C 2 min, 1×: 72°C 10 min, 1×: 4°C forever. The universal Gateway adaptor primers used in the 2^nd ^PCR reactions are following: attB1adaptor_primer: 5'-GGGGACAAGTTTGTACAAAAAAGCAGGCT-3', attB2adaptor_primer: 5'-GGGGACCACTTTGTACAAGAAAGCTGGGT-3'. Five μl from the 2^nd ^PCR reaction is diluted using H_2_O into 25 μl and 1 μl of diluted PCR product is used in the Gateway BP reaction. Gateway BP reactions were done in 96 well plates. Each reaction contains: 1 μl of diluted PCR product, 0.75 μl TE buffer (pH 8.0), 0.25 μl pDONR207 (150 ng/ul), 0.5 μl BP Clonase II mix. The reaction mixture was incubated at 25°C for from 1 hour to overnight depending on the experimental schedule. After incubation, 0.3 μL proteinase K was added to each reaction and incubated at 37°C for 10 min. Transformation was done in 96 well plates as follows: TOP10 competent *E. coli *cells (Invitrogen, Carlsbad, CA) were thawed on ice and 24 μL of cells was added into each BP reaction and incubated on ice for 20 min. Cells were heat shocked at 42°C for 30 sec and put on ice for 2 min, then 175 μL room temp SOC (Invitrogen, Carlsbad, CA) was added to each sample that was then incubated at 37°C, 225 rpm for 1 hour. Fifty μL of each transformation culture was plated on LB plates containing gentamycin (7 μg/mL) using glass beads in 4 Well Rectangular MutiDish w/Lid (Thermo Fisher Scientific, Rochester, NY). For each reaction, two positive colonies were picked and sequenced to confirm the cloned promoter. One μl of DNA from each sequence-confirmed BP clone (robotically isolated in the sequencing center) was used in a Gateway LR reaction using pYXT2 as destination vector in 96 well plates. Each reaction contained: 1 μl of entry clone DNA, 0.75 μl TE buffer (pH 8.0), 0.25 μl pYXT2 (150 ng/μl), 0.5 μl LR Clonase II mix. Incubation time at 25°C was 1 hour to overnight depending on the experimental schedule. LR clone selection was the same as BP clone selection except for the substitution of kanamycin (50 μg/mL) for gentamycin in the LB plates.

### Tri-parental mating with Agrobacterium

For triparental mating, a 50 mL culture of Agrobacterium GV3101 in LB with rifampicin (50 μg/mL) and gentamicin (50 μg/mL) was grown overnight at 28°C, 250 rpm and a 50 mL culture of pRK2013 *E. coli *helper strain in LB with Kan (50 μg/mL) was grown overnight at 37°C, 250 rpm. At the same time, 250 μl cultures of LR clones were incubated overnight in LB medium with kanamycin (50 μg/mL) at 37°C in deep well blocks. The following day, 50 μL Agrobacterium culture, 50 μL *E. coli *helper strain containing pRK2013 plasmid, and 50 μL *E. coli *LR clone culture were plated together on LB agar in each compartment of 4 Well Rectangular MutiDish w/Lid (Thermo Fisher Scientific, Rochester, NY) for each LR clone. The following day, from the bacterial lawn, a small loopful of bacteria from each well was streaked onto LB plates containing rifampicin (50 μg/mL), gentamicin (50 μg/mL) and kanamycin (50 μg/mL) to select for Agrobacterium with the pYXT2 construct containing the target promoters. Two positive colonies from each reaction were inoculated into corresponding positions of two 96 well blocks with 250 μL LB media containing rifampicin (50 μg/mL), gentamicin (50 μg/mL), kanamycin (50 μg/mL) and grown at 28°C, 225 rpm for 2 days. PCR was then used to confirm the positively selected Agrobacterium colonies as follows: Twenty μL of 20 mM NaOH solution were added into a new PCR plate, then 3 μL Agrobacterium culture were added into each well. The PCR plate was sealed and incubated at 37°C for 5 minutes. Then 2 μL of NaOH treated Agrobacterium culture were transferred into new PCR plate and a PCR reaction was set up by adding 4.0 μL H_2_O, 10 μL 2× PCR mix (New England Bio Lab, Ipswich, MA), 4.0 μL promoter-specific primer pair (2.5 μM). The PCR conditions are: 1×: 95°C 2 min, 30×: 94°C 30 sec, 55°C 30 sec, 72°C 3 min, 1×: 72°C 5 min, 1×: 4°C forever. The positive colonies were used in plant transformation.

### Transformation and Plant Growth

*Arabidopsis thaliana *ecotype Columbia-0 was used in all our experiments. Plants were grown on Redimix at 25°C and 24 hours light (cool white fluorescent; ~150 microeinsteins). Plant transformation and seed selection was done according to standard methods [35] except that only 50 ml of the Agrobacterium was cultured and used in floral dipping so that all subsequent manipulations up to and including floral dipping were done in 50 ml Falcon tubes (Becton Dickson Labware, Franklin Lakes, NJ). Three independent transformations were done for each construct (i.e. three plants were dipped separately in the same Agrobacterium tube) and up to 3 plants from each kanamycin (50 μg/mL) selection plate were transferred into soil.

### Microscopy

Plants were observed using an Olympus SZX12 stereomicroscope equipped with a 100 W mercury lamp for epifluorescence and a parfocal 1.6× objective. Images were recorded with an Olympus DP71 digital camera.

### Quantitative real-time PCR

Leaf RNA was isolated from Plant basal rosette leaves from plants about 21 days after germination. Flower RNA was isolated opened flowers and unopened flower buds. Young silique RNA was isolated from immature siliques; root RNA isolation has been described previously [61]. RNA was extracted using TRIzol (Invitrogen, Carlsbad, CA, USA) as described by the manufacturer and then filtered using RNeasy columns (Qiagen, Valencia, CA, USA). First-strand cDNA was synthesized by priming with oligo-dT using SuperScript III reverse transcriptase (Invitrogen Carlsbad, CA, USA) following the instructions of the provider. PCR reactions were carried out in an ABI PRISM^® ^7900 HT Sequence Detection System (Applied Biosystems, Foster City, CA, USA). SYBR^® ^Green was used to quantify dsDNA synthesis. Reactions (10 μl total volume) were amplified using the following standard PCR protocol: 50°C for 2 min; 95°C for 10 min; 40 cycles of 95°C for 15 sec and 60°C for 1 min, and SYBR^® ^Green fluorescence was measured continuously. Three biological and 2 technical replicates were used for analysis. PCR efficiency was estimated using LinReg software with data obtained from the exponential phase of each individual amplification plot. Cycle time (Ct) values were taken at a threshold value of 0.2.

## Competing interests

The authors declare that they have no competing interests.

## Authors' contributions

YX participated in the experimental work and wrote the manuscript. JCR, ELM, BAU, WAM, and WW performed the experiments. JZ and HCW carried out bioinformatic and database-related tasks. CDT conceived and coordinated the project and contributed to the manuscript.

All authors have read and approved the final manuscript.

## Supplementary Material

Additional file 1 **Table S1**. Identifiers and promoter sequences og genes used in this study. Click here for file

Additional file 2 **Table S2**. Listing of all genes used in this study with their TAIR identifiers (where available) and the success status at each step in the transgenic plant production pipeline. Click here for file

Additional file 3 **Table S3**. Listing of all genes assigned to each Plant Ontology (PO) code. Click here for file

Additional file 4 **Table S4**. Validation of GFP expression patterns by quantitative real time RT-PCR. Click here for file

## References

[B1] Initiative AG Analysis of the genome sequence of the flowering plant Arabidopsis thaliana Nature 2000 408 796 815 10.1038/35048692 11130711

[B2] Chory J Ecker JR Briggs S Caboche M Coruzzi GM Cook D Dangl J Grant S Guerinot ML Henikoff S National Science Foundation-Sponsored Workshop Report: "The 2010 Project" functional genomics and the virtual plant. A blueprint for understanding how plants are built and how to improve them Plant Physiol 2000 123 423 426 10.1104/pp.123.2.423 10859172 PMC1539254

[B3] Wisman E Ohlrogge J Arabidopsis microarray service facilities Plant Physiol 2000 124 1468 1471 10.1104/pp.124.4.1468 11115861 PMC1539298

[B4] Zhu T Wang X Large-scale profiling of the Arabidopsis transcriptome Plant Physiol 2000 124 1472 1476 10.1104/pp.124.4.1472 11115862 PMC1539299

[B5] Redman JC Haas BJ Tanimoto G Town CD Development and evaluation of an Arabidopsis whole genome Affymetrix probe array Plant J 2004 38 545 561 10.1111/j.1365-313X.2004.02061.x 15086809

[B6] Kim H Snesrud EC Haas B Cheung F Town CD Quackenbush J Gene expression analyses of Arabidopsis chromosome 2 using a genomic DNA amplicon microarray Genome Res 2003 13 327 340 10.1101/gr.552003 12618363 PMC430289

[B7] Yamada K Lim J Dale JM Chen H Shinn P Palm CJ Southwick AM Wu HC Kim C Nguyen M Empirical analysis of transcriptional activity in the Arabidopsis genome Science 2003 302 842 846 10.1126/science.1088305 14593172

[B8] Stolc V Samanta MP Tongprasit W Sethi H Liang S Nelson DC Hegeman A Nelson C Rancour D Bednarek S Identification of transcribed sequences in Arabidopsis thaliana by using high-resolution genome tiling arrays Proc Natl Acad Sci USA 2005 102 4453 4458 10.1073/pnas.0408203102 15755812 PMC555476

[B9] Laubinger S Zeller G Henz SR Sachsenberg T Widmer CK Naouar N Vuylsteke M Scholkopf B Ratsch G Weigel D At-TAX: a whole genome tiling array resource for developmental expression analysis and transcript identification in Arabidopsis thaliana Genome Biol 2008 9 R112 10.1186/gb-2008-9-7-r112 18613972 PMC2530869

[B10] Schubeler D MacAlpine DM Scalzo D Wirbelauer C Kooperberg C van Leeuwen F Gottschling DE O'Neill LP Turner BM Delrow J The histone modification pattern of active genes revealed through genome-wide chromatin analysis of a higher eukaryote Genes Dev 2004 18 1263 1271 10.1101/gad.1198204 15175259 PMC420352

[B11] Gendrel AV Lippman Z Martienssen R Colot V Profiling histone modification patterns in plants using genomic tiling microarrays Nat Methods 2005 2 213 218 10.1038/nmeth0305-213 16163802

[B12] Kurdistani SK Tavazoie S Grunstein M Mapping global histone acetylation patterns to gene expression Cell 2004 117 721 733 10.1016/j.cell.2004.05.023 15186774

[B13] Chang S Pikaard CS Transcript profiling in Arabidopsis reveals complex responses to global inhibition of DNA methylation and histone deacetylation J Biol Chem 2005 280 796 804 15516340 10.1074/jbc.M409053200

[B14] Ren B Robert F Wyrick JJ Aparicio O Jennings EG Simon I Zeitlinger J Schreiber J Hannett N Kanin E Genome-wide location and function of DNA binding proteins Science 2000 290 2306 2309 10.1126/science.290.5500.2306 11125145

[B15] Thibaud-Nissen F Wu H Richmond T Redman JC Johnson C Green R Arias J Town CD Development of Arabidopsis whole-genome microarrays and their application to the discovery of binding sites for the TGA2 transcription factor in salicylic acid-treated plants Plant J 2006 47 152 162 10.1111/j.1365-313X.2006.02770.x 16824183

[B16] Springer PS Gene traps: tools for plant development and genomics Plant Cell 2000 12 1007 1020 10.1105/tpc.12.7.1007 10899970 PMC149045

[B17] Li HM Altschmied L Chory J Arabidopsis mutants define downstream branches in the phototransduction pathway Genes Dev 1994 8 339 349 10.1101/gad.8.3.339 8314087

[B18] Manners JM Penninckx IA Vermaere K Kazan K Brown RL Morgan A Maclean DJ Curtis MD Cammue BP Broekaert WF The promoter of the plant defensin gene PDF1.2 from Arabidopsis is systemically activated by fungal pathogens and responds to methyl jasmonate but not to salicylic acid Plant Mol Biol 1998 38 1071 1080 10.1023/A:1006070413843 9869413

[B19] Santamaria M Thomson CJ Read ND Loake GJ The promoter of a basic PR1-like gene, AtPRB1, from Arabidopsis establishes an organ-specific expression pattern and responsiveness to ethylene and methyl jasmonate Plant Mol Biol 2001 47 641 652 10.1023/A:1012410009930 11725949

[B20] Vicente-Agullo F Rigas S Desbrosses G Dolan L Hatzopoulos P Grabov A Potassium carrier TRH1 is required for auxin transport in Arabidopsis roots Plant J 2004 40 523 535 10.1111/j.1365-313X.2004.02230.x 15500468

[B21] Peiter E Montanini B Gobert A Pedas P Husted S Maathuis FJ Blaudez D Chalot M Sanders D A secretory pathway-localized cation diffusion facilitator confers plant manganese tolerance Proc Natl Acad Sci USA 2007 104 8532 8537 10.1073/pnas.0609507104 17494768 PMC1895984

[B22] Bernal AJ Yoo CM Mutwil M Jensen JK Hou G Blaukopf C Sorensen I Blancaflor EB Scheller HV Willats WG Functional analysis of the cellulose synthase-like genes CSLD1, CSLD2, and CSLD4 in tip-growing Arabidopsis cells Plant Physiol 2008 148 1238 1253 10.1104/pp.108.121939 18768911 PMC2577265

[B23] Hong RL Hamaguchi L Busch MA Weigel D Regulatory elements of the floral homeotic gene AGAMOUS identified by phylogenetic footprinting and shadowing Plant Cell 2003 15 1296 1309 10.1105/tpc.009548 12782724 PMC156367

[B24] Larkin JC Oppenheimer DG Pollock S Marks MD Arabidopsis GLABROUS1 Gene Requires Downstream Sequences for Function Plant Cell 1993 5 1739 1748 10.1105/tpc.5.12.1739 12271054 PMC160400

[B25] Stam M Belele C Dorweiler JE Chandler VL Differential chromatin structure within a tandem array 100 kb upstream of the maize b1 locus is associated with paramutation Genes Dev 2002 16 1906 1918 10.1101/gad.1006702 12154122 PMC186425

[B26] Moskal WA Jr Wu HC Underwood BA Wang W Town CD Xiao Y Experimental validation of novel genes predicted in the un-annotated regions of the Arabidopsis genome BMC Genomics 2007 8 18 10.1186/1471-2164-8-18 17229318 PMC1783852

[B27] Schiex TMA Rouzé P EuGene: an eukaryotic gene finder that combines several sources of evidence Lect Notes in Comput Sci 2006 11 125

[B28] Korf I Flicek P Duan D Brent MR Integrating genomic homology into gene structure prediction Bioinformatics 2001 17 Suppl 1 S140 148 11473003 10.1093/bioinformatics/17.suppl_1.s140

[B29] Xiao YL Smith SR Ishmael N Redman JC Kumar N Monaghan EL Ayele M Haas BJ Wu HC Town CD Analysis of the cDNAs of hypothetical genes on Arabidopsis chromosome 2 reveals numerous transcript variants Plant Physiol 2005 139 1323 1337 10.1104/pp.105.063479 16244158 PMC1283769

[B30] Nakano M Nobuta K Vemaraju K Tej SS Skogen JW Meyers BC Plant MPSS databases: signature-based transcriptional resources for analyses of mRNA and small RNA Nucleic Acids Res 2006 34 D731 735 10.1093/nar/gkj077 16381968 PMC1347440

[B31] Underwood BA Vanderhaeghen R Whitford R Town CD Hilson P Simultaneous high-throughput recombinational cloning of open reading frames in closed and open configurations Plant Biotechnol J 2006 4 317 324 10.1111/j.1467-7652.2006.00183.x 17147637

[B32] Hartley JL Temple GF Brasch MA DNA cloning using in vitro site-specific recombination Genome Res 2000 10 1788 1795 10.1101/gr.143000 11076863 PMC310948

[B33] Van Haute E Joos H Maes M Warren G Van Montagu M Schell J Intergeneric transfer and exchange recombination of restriction fragments cloned in pBR322: a novel strategy for the reversed genetics of the Ti plasmids of Agrobacterium tumefaciens Embo J 1983 2 411 417 11894957 10.1002/j.1460-2075.1983.tb01438.x PMC555148

[B34] Xiao YL Malik M Whitelaw CA Town CD Cloning and sequencing of cDNAs for hypothetical genes from chromosome 2 of Arabidopsis Plant Physiol 2002 130 2118 2128 10.1104/pp.010207 12481096 PMC166724

[B35] Clough SJ Bent AF Floral dip: a simplified method for Agrobacterium-mediated transformation of Arabidopsis thaliana Plant J 1998 16 735 743 10.1046/j.1365-313x.1998.00343.x 10069079

[B36] Jaiswal P Avraham S Ilic K Kellogg EA McCouch S Pujar A Reiser L Rhee SY Sachs MM Schaeffer M Plant Ontology (PO): a Controlled Vocabulary of Plant Structures and Growth Stages Comp Funct Genomics 2005 6 388 397 10.1002/cfg.496 18629207 PMC2447502

[B37] Klimyuk VI Carroll BJ Thomas CM Jones JD Alkali treatment for rapid preparation of plant material for reliable PCR analysis Plant J 1993 3 493 494 10.1046/j.1365-313X.1993.t01-26-00999.x 8220456

[B38] Bougourd S Marrison J Haseloff J Technical advance: an aniline blue staining procedure for confocal microscopy and 3 D imaging of normal and perturbed cellular phenotypes in mature Arabidopsis embryos Plant J 2000 24 543 550 10.1046/j.1365-313x.2000.00892.x 11115135

[B39] de Boer GJ Testerink C Pielage G Nijkamp HJ Stuitje AR Sequences surrounding the transcription initiation site of the Arabidopsis enoyl-acyl carrier protein reductase gene control seed expression in transgenic tobacco Plant Mol Biol 1999 39 1197 1207 10.1023/A:1006129924683 10380806

[B40] McKinney EC Kandasamy MK Meagher RB Small changes in the regulation of one Arabidopsis profilin isovariant, PRF1, alter seedling development Plant Cell 2001 13 1179 1191 10.1105/tpc.13.5.1179 11340190 PMC135555

[B41] Stone JM Liang X Nekl ER Stiers JJ Arabidopsis AtSPL14, a plant-specific SBP-domain transcription factor, participates in plant development and sensitivity to fumonisin B1 Plant J 2005 41 744 754 10.1111/j.1365-313X.2005.02334.x 15703061

[B42] Welchen E Gonzalez DH Differential expression of the Arabidopsis cytochrome c genes Cytc-1 and Cytc-2. Evidence for the involvement of TCP-domain protein-binding elements in anther- and meristem-specific expression of the Cytc-1 gene Plant Physiol 2005 139 88 100 10.1104/pp.105.065920 16113211 PMC1203360

[B43] Magnan F Ranty B Charpenteau M Sotta B Galaud JP Aldon D Mutations in AtCML9, a calmodulin-like protein from Arabidopsis thaliana, alter plant responses to abiotic stress and abscisic acid Plant J 2008 56 575 589 10.1111/j.1365-313X.2008.03622.x 18643966

[B44] Mills RF Doherty ML Lopez-Marques RL Weimar T Dupree P Palmgren MG Pittman JK Williams LE ECA3, a Golgi-localized P2A-type ATPase, plays a crucial role in manganese nutrition in Arabidopsis Plant Physiol 2008 146 116 128 10.1104/pp.107.110817 18024560 PMC2230566

[B45] Wise AA Liu Z Binns AN Three methods for the introduction of foreign DNA into Agrobacterium Methods Mol Biol 2006 343 43 53 16988332 10.1385/1-59745-130-4:43

[B46] Davis AM Hall A Millar AJ Darrah C Davis SJ Protocol: Streamlined sub-protocols for floral-dip transformation and selection of transformants in Arabidopsis thaliana Plant Methods 2009 5 3 10.1186/1746-4811-5-3 19250520 PMC2660325

[B47] Desfeux C Clough SJ Bent AF Female reproductive tissues are the primary target of Agrobacterium-mediated transformation by the Arabidopsis floral-dip method Plant Physiol 2000 123 895 904 10.1104/pp.123.3.895 10889238 PMC59052

[B48] Chilton MD Que Q Targeted integration of T-DNA into the tobacco genome at double-stranded breaks: new insights on the mechanism of T-DNA integration Plant Physiol 2003 133 956 965 10.1104/pp.103.026104 14551336 PMC281593

[B49] Francis KE Spiker S Identification of Arabidopsis thaliana transformants without selection reveals a high occurrence of silenced T-DNA integrations Plant J 2005 41 464 477 10.1111/j.1365-313X.2004.02312.x 15659104

[B50] Sutton T Baumann U Hayes J Collins NC Shi BJ Schnurbusch T Hay A Mayo G Pallotta M Tester M Langridge P Boron-toxicity tolerance in barley arising from efflux transporter amplification Science 2007 318 1446 1449 10.1126/science.1146853 18048688

[B51] Morel M Crouzet J Gravot A Auroy P Leonhardt N Vavasseur A Richaud P AtHMA3, a P1B-ATPase allowing Cd/Zn/Co/Pb vacuolar storage in Arabidopsis Plant Physiol 2009 149 894 904 10.1104/pp.108.130294 19036834 PMC2633814

[B52] Bailey TL Elkan C Fitting a mixture model by expectation maximization to discover motifs in biopolymers Proc Int Conf Intell Syst Mol Biol 1994 2 28 36 7584402

[B53] Cho SK Larue CT Chevalier D Wang H Jinn TL Zhang S Walker JC Regulation of floral organ abscission in Arabidopsis thaliana Proc Natl Acad Sci USA 2008 105 15629 15634 10.1073/pnas.0805539105 18809915 PMC2563077

[B54] McKim SM Stenvik GE Butenko MA Kristiansen W Cho SK Hepworth SR Aalen RB Haughn GW The BLADE-ON-PETIOLE genes are essential for abscission zone formation in Arabidopsis Development 2008 135 1537 1546 10.1242/dev.012807 18339677

[B55] Perez-Amador MA Abler ML De Rocher EJ Thompson DM van Hoof A LeBrasseur ND Lers A Green PJ Identification of BFN1, a bifunctional nuclease induced during leaf and stem senescence in Arabidopsis Plant Physiol 2000 122 169 180 10.1104/pp.122.1.169 10631260 PMC58855

[B56] Farage-Barhom S Burd S Sonego L Perl-Treves R Lers A Expression analysis of the BFN1 nuclease gene promoter during senescence, abscission, and programmed cell death-related processes J Exp Bot 2008 59 3247 3258 10.1093/jxb/ern176 18603613 PMC2529240

[B57] Cai S Lashbrook CC Stamen abscission zone transcriptome profiling reveals new candidates for abscission control: enhanced retention of floral organs in transgenic plants overexpressing Arabidopsis ZINC FINGER PROTEIN2 Plant Physiol 2008 146 1305 1321 10.1104/pp.107.110908 18192438 PMC2259061

[B58] Liu Z Liu Z The second intron of AGAMOUS drives carpel- and stamen-specific expression sufficient to induce complete sterility in Arabidopsis Plant Cell Rep 2008 27 855 863 10.1007/s00299-008-0511-3 18256838

[B59] Bhalla PL Swoboda I Singh MB Reduction in allergenicity of grass pollen by genetic engineering Int Arch Allergy Immunol 2001 124 51 54 10.1159/000053666 11306924

[B60] Altschul SF Gish W Miller W Myers EW Lipman DJ Basic local alignment search tool J Mol Biol 1990 215 403 410 2231712 10.1016/S0022-2836(05)80360-2

[B61] Xiao YL Malik M Whitelaw CA Town CD Cloning and sequencing of cDNAs for hypothetical genes from chromosome 2 of Arabidopsis Plant Physiol 2002 130 2118 28 10.1104/pp.010207 12481096 PMC166724

