# Supplementary material for: High throughput generation of promoter reporter (GFP) transgenic lines of low expressing genes in Arabidopsis and analysis of their expression patterns
Source: Plant Methods. 2010 Aug 6;6:18. doi: 10.1186/1746-4811-6-18 (PMC2927586; doi:10.1186/1746-4811-6-18)
Supplement: Additional file 3 — Table S3. Listing of all genes assigned to each Plant Ontology (PO) code. [file 1746-4811-6-18-S3.DOC]

Table S3. Listing of genes assigned to each Plant Ontology code

PO:0000036 At.chr1.16.7 At.chr1.17.278 AT.CHR1.19.84 At.chr1.20.34 AT.CHR4.7.322 At03eug23420 At05eug36530 At05eug49430 AT1G01530 AT1G11250 AT1G18350 AT1G25340 AT1G49270 AT1G58120 AT1G73560 AT1G77200 AT1G80580 AT2G22760 AT2G33260 AT2G42480 AT2G47050 AT3G14880 AT3G20865 AT3G53060 AT3G58780 AT4G29650 AT4G30830 AT4G35280 AT4G37780 AT4G39590 AT5G02640 AT5G14980 AT5G17080 AT5G26930 AT5G58360 AT5G58680 AT5G62320 novel_chr1_5915494

PO:0009005 At.chr1.20.34 At01eug36840 At03eug44940 At05eug26140 AT1G01530 AT1G07460 AT1G21530 AT1G30740 AT1G47220 AT1G49270 AT1G55640 AT1G56030 AT1G64820 AT1G77200 AT1G77405 AT1G80580 AT2G28680 AT3G53060 AT3G66656 AT4G02300 AT4G18770 AT4G30830 AT4G37780 AT5G02070 AT5G23650 AT5G26930 AT5G27910 AT5G45810 AT5G53520 AT5G54000 AT5G57810 AT5G58360 AT5G58680 AT5G58850 AT5G60080 AT5G60110 novel_chr1_5915494

PO:0005660 At.Chr1.15.120 AT.CHR1.19.84 AT.CHR3.14.320 AT.CHR4.5.258 AT.CHR4.7.322 At01eug36840 At02eug13430 At03eug31320 AT1G01530 AT1G06020 AT1G11250 AT1G18350 AT1G35730 AT1G55640 AT1G80580 AT2G19980 AT2G33260 AT2G34440 AT2G47050 AT4G00416 AT4G30830 AT4G37780 AT5G15250 AT5G16500 AT5G23650 AT5G35090 AT5G45810 AT5G51860 AT5G53520 AT5G54000 AT5G58360 AT5G58850 AT5G60110 AT5G62320 novel_chr1_5915494

PO:0000013 At.chr1.20.34 AT.CHR4.7.322 At01eug36840 AT1G02710 AT1G18350 AT1G35730 AT1G55640 AT1G56360 AT1G58120 AT1G64820 AT1G77200 AT1G77655 AT2G03130 AT2G22760 AT2G33260 AT2G40250 AT3G42830 AT3G56700 AT3G58780 AT4G00416 AT4G18395 AT4G35280 AT4G37780 AT5G02640 AT5G11080 AT5G52770 AT5G53520 AT5G54000 AT5G58850 AT5G60110 AT5G62320 novel_chr1_5915494

PO:0003011 AT.CHR4.7.322 At03eug31320 AT1G02710 AT1G11370 AT1G24110 AT1G25340 AT1G29490 AT1G33930 AT1G48800 AT2G17940 AT2G24140 AT2G42480 AT3G14880 AT3G23130 AT3G24130 AT3G24680 AT3G45280 AT3G58780 AT4G13985 AT4G29650 AT4G30830 AT4G35280 AT4G37780 AT5G02640 AT5G13350 AT5G14980 AT5G24820 AT5G27910 AT5G58360 AT5G58680 AT5G66970 novel_chr1_5915494

PO:0000056 AT.CHR4.7.322 At03eug23420 AT1G11250 AT1G17310 AT1G32250 AT1G35730 AT1G51960 AT1G77200 AT2G17845 AT2G21420 AT2G22760 AT2G40250 AT3G45280 AT3G56700 AT3G58780 AT3G60020 AT4G10260 AT4G14815 AT4G35280 AT4G37780 AT5G11080 AT5G17080 AT5G24820 AT5G28470 AT5G62320 novel_chr1_5915494

PO:0006504 AT.CHR1.19.84 At.chr1.20.34 AT.CHR2.4.139 At05eug26140 AT1G02710 AT1G21530 AT1G25340 AT1G33930 AT1G53620 AT1G64820 AT1G73560 AT1G77405 AT2G17845 AT2G17940 AT2G21420 AT2G22760 AT2G28610 AT2G28680 AT2G40250 AT3G56700 AT4G13985 AT4G18770 AT4G30830 AT5G58850 novel_chr1_5915494

PO:0009052 AT.CHR2.4.139 AT.CHR4.2.212 AT.CHR4.7.322 At01eug36840 At05eug36530 AT1G17310 AT1G35730 AT1G69500 AT2G37030 AT2G40250 AT2G47050 AT3G14880 AT3G42830 AT3G58780 AT4G00416 AT4G21690 AT4G35280 AT5G02640 AT5G11080 AT5G28520 AT5G51860 AT5G53520 AT5G58680 novel_chr1_5915494

PO:0009032 AT.CHR2.4.139 AT.CHR4.7.322 AT.CHR4.8.391 At03eug23420 At03eug23510 AT1G17310 AT1G18350 AT1G21530 AT1G25340 AT1G32250 AT1G53620 AT1G77200 AT2G07070 AT2G17845 AT2G17940 AT2G28610 AT2G40250 AT3G45940 AT3G56700 AT4G30830 AT5G17080 novel_chr1_5915494

PO:0006502 AT.CHR4.2.212 AT.CHR4.8.391 At.chr5.3.34 At05eug26140 AT1G02710 AT1G51960 AT1G70720 AT1G77655 AT2G03130 AT2G28680 AT3G23130 AT4G18395 AT4G21690 AT5G02640 AT5G11080 AT5G15250 AT5G28470 AT5G45810 AT5G46370 AT5G54000 novel_chr1_5915494

PO:0006325 At.chr1.20.34 AT.CHR4.2.212 AT.CHR4.8.391 At01eug36840 AT1G35730 AT1G51960 AT1G70720 AT1G77200 AT2G03130 AT2G30370 AT3G56700 AT4G11510 AT4G21690 AT4G37780 AT5G02640 AT5G11080 AT5G24820 AT5G28470 AT5G60110 novel_chr1_5915494

PO:0000282 AT.CHR1.19.84 At.chr1.20.34 AT.CHR1.8.296 AT.CHR2.4.139 At.chr4.5.106 At03eug13620 AT1G02710 AT1G21530 AT1G25340 AT1G53620 AT1G64820 AT2G21420 AT2G40250 AT3G56700 AT4G18770 AT4G30830 AT5G16500 AT5G54000 AT5G58360 AT5G58850

PO:0009031 At.chr1.20.34 AT.CHR2.4.139 At03eug23510 At03eug25870 AT1G25340 AT1G69500 AT2G21420 AT2G28610 AT3G45940 AT3G58780 AT4G00416 AT4G18770 AT4G21690 AT5G02640 AT5G52770 AT5G53520 AT5G54000 AT5G58850 novel_chr1_5915494

PO:0000054 At.chr1.16.7 At.chr1.17.278 AT.CHR4.2.212 AT.CHR4.8.391 At03eug23420 At05eug26140 At05eug36530 AT1G18350 AT1G69500 AT2G28610 AT3G58780 AT4G21690 AT4G30830 AT4G39590 AT5G02640 AT5G62850 novel_chr1_5915494

PO:0009001 AT.CHR2.4.139 AT.CHR4.7.322 AT1G25340 AT2G40250 AT2G47050 AT3G56700 AT4G00416 AT4G21690 AT5G02640 AT5G11080 AT5G24820 AT5G51860 AT5G53520 novel_chr1_5915494

PO:0020139 At03eug23420 AT1G11370 AT1G18350 AT1G77655 AT2G03130 AT2G17940 AT2G42480 AT3G45280 AT3G58780 AT4G13985 AT4G21690 AT4G30830 AT5G11080 AT5G26930

PO:0000293 AT.CHR1.19.84 AT.CHR3.14.320 At01eug36840 AT1G25340 AT1G31080 AT2G20660 AT3G53060 AT4G00416 AT4G21690 AT5G02640 AT5G51860 AT5G53520

PO:0009049 At.chr1.20.34 AT.CHR4.7.322 AT1G02710 AT1G18530 AT2G22760 AT2G40250 AT3G56700 AT4G00416 AT4G21690 AT5G51860 AT5G58680 novel_chr1_5915494

PO:0009047 AT.CHR4.7.322 AT1G02710 AT1G18530 AT2G22760 AT3G58780 AT4G00416 AT4G21690 AT5G02640 AT5G51860 AT5G53520 AT5G58680 novel_chr1_5915494

PO:0006056 At01eug36840 AT1G06020 AT1G77405 AT2G17940 AT2G20660 AT2G28680 AT3G45280 AT3G56700 AT5G58680 AT5G58850 novel_chr1_5915494

PO:0006016 At01eug36840 AT1G06020 AT1G11370 AT1G77405 AT2G20660 AT2G22760 AT4G37780 AT5G15250 AT5G52770 AT5G58680 novel_chr1_5915494

PO:0000112 AT.CHR4.7.322 At03eug31320 AT2G22760 AT2G40250 AT3G14880 AT3G56700 AT3G58780 AT5G24820 AT5G52770 AT5G58680 novel_chr1_5915494

PO:0000115 AT.CHR1.19.84 AT1G17310 AT1G21530 AT1G48800 AT3G23130 AT3G56700 AT4G13985 AT4G37780 AT5G58360 AT5G58850

PO:0000146 At.chr5.3.34 AT1G17310 AT1G48800 AT1G51960 AT2G28680 AT2G30370 AT3G23130 AT4G13985 AT5G28470

PO:0004707 AT.CHR4.1.217 AT1G25340 AT1G35730 AT1G77200 AT2G22760 AT3G58780 AT4G30830 AT4G37780 AT5G02640

PO:0009010 At.chr1.20.34 AT1G18350 AT1G77200 AT2G02515 AT3G42830 AT3G58780 AT5G14980 AT5G39280 novel_chr1_5915494

PO:0004723 At.chr1.16.7 At.chr1.17.278 AT.CHR4.2.212 At05eug36530 AT1G35730 AT1G69500 AT4G30830 AT5G02640 AT5G58360

PO:0009067 AT1G02710 AT1G48800 AT1G77200 AT2G17845 AT2G40250 AT3G58780 AT4G21200 novel_chr1_5915494

PO:0020127 AT1G17310 AT1G53620 AT2G22760 AT2G31760 AT3G05720 AT4G28090 AT5G28520 AT5G38195

PO:0006040 AT2G22760 AT2G37030 AT2G40250 AT2G47050 AT4G37780 AT5G11080 AT5G58850 novel_chr1_5915494

PO:0000039 At03eug31320 AT1G29490 AT1G48800 AT1G58120 AT1G77200 AT3G58780 AT4G30830 AT5G62380

PO:0000034 AT.CHR1.19.84 AT.CHR3.14.320 AT.CHR4.5.258 At03eug23420 At05eug49430 AT1G77200 AT5G58360 novel_chr1_5915494

PO:0006501 AT1G51960 AT1G77655 AT2G03130 AT4G18395 AT4G21690 AT5G54000 AT5G58850

PO:0020128 At05eug36530 AT1G77200 AT2G17940 AT2G22760 AT2G28610 AT2G28680 AT4G28090

PO:0000332 AT2G03130 AT2G37030 AT3G56700 AT4G37780 AT5G02640 AT5G37560 AT5G58850

PO:0005003 At.chr1.20.34 AT1G64820 AT2G21420 AT3G56700 AT4G30830 AT5G15250 AT5G58850

PO:0000035 AT1G18280 AT2G47050 AT3G58780 AT4G29650 AT4G35280 AT5G58360

PO:0005679 At05eug26140 AT1G77200 AT4G21690 AT4G37780 AT5G11080 AT5G24820

PO:0009025 AT1G31080 AT3G23130 AT4G13985 AT4G37780 AT5G37560 AT5G51860

PO:0006036 AT1G53620 AT1G77405 AT2G22760 AT2G43890 AT4G13985 AT5G26930

PO:0009030 AT.CHR2.4.139 AT.CHR4.1.217 AT.CHR4.2.212 AT.CHR4.7.322 novel_chr1_5915494

PO:0000025 AT.CHR4.7.322 AT1G64820 AT3G24680 AT4G13985 AT5G45810

PO:0009015 AT.CHR1.19.84 AT2G33260 AT4G35280 AT4G37780 AT5G11080

PO:0020030 AT1G17310 AT1G48800 AT1G55640 AT4G37780

PO:0004536 AT1G69500 AT3G56700 AT4G13985 AT4G37780

PO:0009072 AT1G35730 AT1G77200 AT2G40250 AT5G51860

PO:0000052 AT1G17310 AT2G42480 AT4G37780 AT5G26930

PO:0000256 AT1G55640 AT1G61950 AT1G64820 AT5G60110

PO:0009046 At03eug23420 AT1G35730 AT3G58780

PO:0004724 AT1G48800 AT1G80580 AT2G28680

PO:0009081 AT1G17310 AT2G22760 AT5G58680

PO:0005028 AT1G18350 AT4G30830 AT5G26930

PO:0005645 At05eug49430 AT2G31760 AT3G58780

PO:0009053 AT2G22760 AT3G58780 AT5G24820

PO:0005021 AT1G17310 AT2G17940 AT2G28610

PO:0000033 AT.CHR4.7.322 AT1G77200 AT2G22760

PO:0005011 AT1G02710 AT5G17080

PO:0005008 AT1G58120 AT3G45280

PO:0008003 AT1G58120 AT5G26930

PO:0020100 AT1G80580 AT2G28680

PO:0006019 AT1G18350 AT2G37030

PO:0004006 At05eug49430 AT2G22760

PO:0000051 AT2G22760 AT4G13985

PO:0020031 AT2G28680 AT5G46370

PO:0020141 At.Chr1.15.120 AT1G11250

PO:0004711 AT1G51960

PO:0005019 At.chr1.16.7

PO:0006338 AT.CHR4.7.322

PO:0008015 AT4G29650

PO:0006339 AT.CHR4.7.322

PO:0020121 AT5G51860

PO:0000017 AT2G31760

PO:0006034 AT2G31760

PO:0020091 AT2G24370

PO:0005012 AT1G58120

PO:0006041 AT2G47050

PO:0006081 AT4G21200

PO:0006085 AT1G31080

PO:0000014 AT2G31760

PO:0009073 AT1G35730

PO:0020041 At01eug36840
