# Supplementary material for: High throughput generation of promoter reporter (GFP) transgenic lines of low expressing genes in Arabidopsis and analysis of their expression patterns
Source: Plant Methods. 2010 Aug 6;6:18. doi: 10.1186/1746-4811-6-18 (PMC2927586; doi:10.1186/1746-4811-6-18)
Supplement: Additional file 4 — Table S4. Validation of GFP expression patterns by quantitative real time RT-PCR. [file 1746-4811-6-18-S4.DOC]

Table S4. Confirmation of GFP expression patterns by quantitative real time RT-PCR

| Gene name | Promoter-GFP localization |  | Expression levels in various tissues determined by quantitative RT-PCR*. | | | |
| --- | --- | --- | --- | --- | --- | --- |
|  |  |  | Flower | Leaf | Root | Young silique |
| AT1G11250.1 | Floral buds | 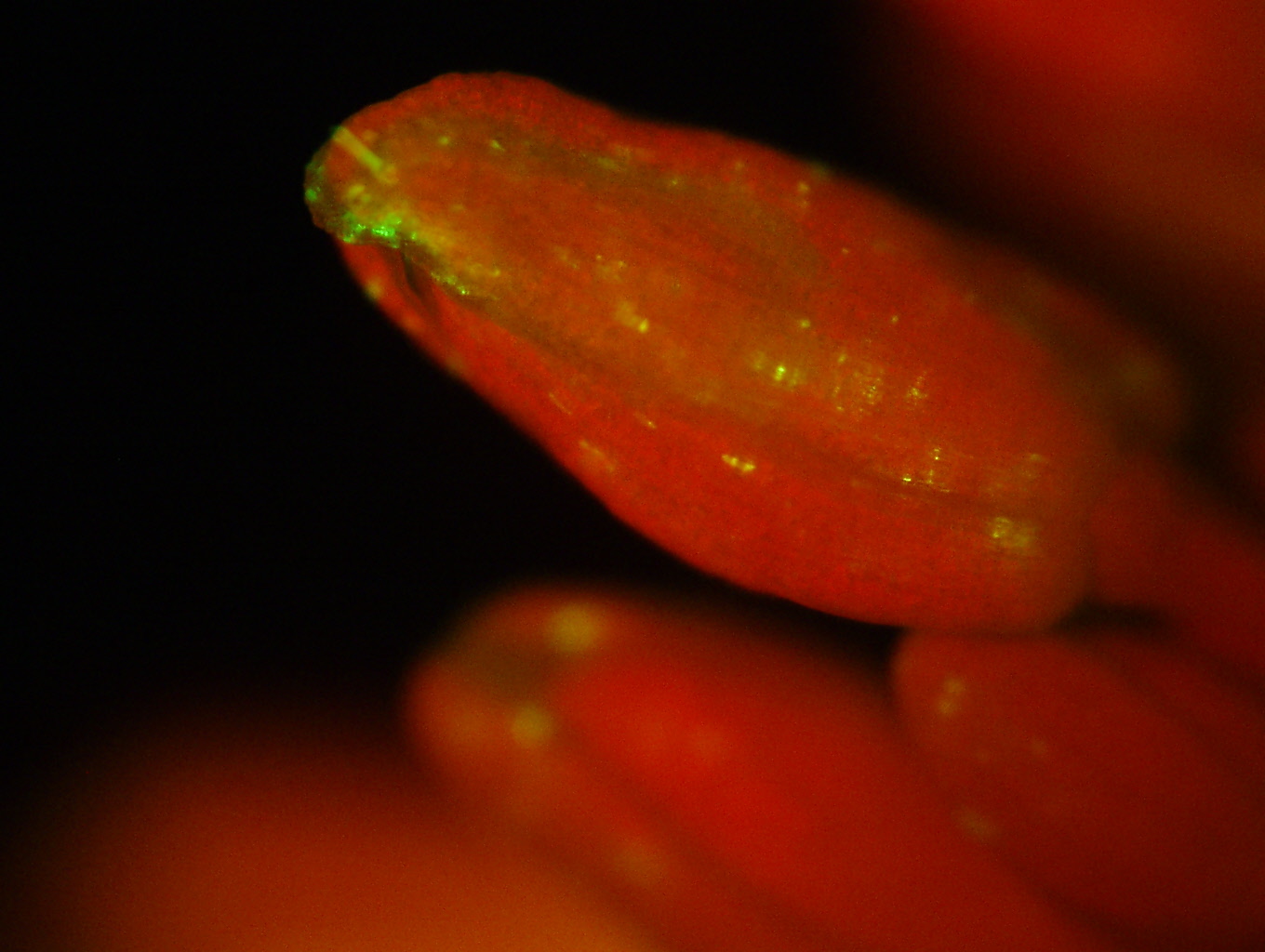 | 23.5 ± 0.6 | 32.5 ± 1.6 | 29.9 ± 0.2 | 25.1 ± 0.2 |
| AT1G48800.1 | Roots | 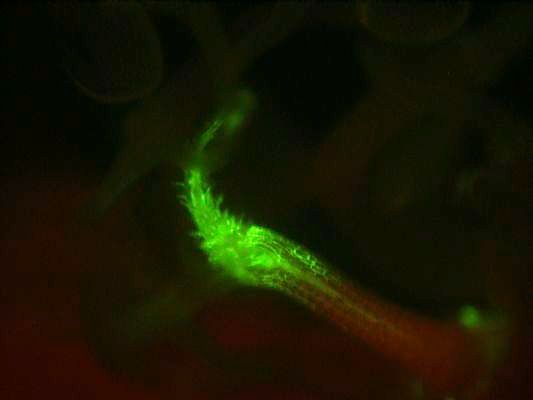 | 34.0 ± 0.6 | ND† | 22.9 ± 0.4 | ND |
| AT1G53620.1 | Roots | 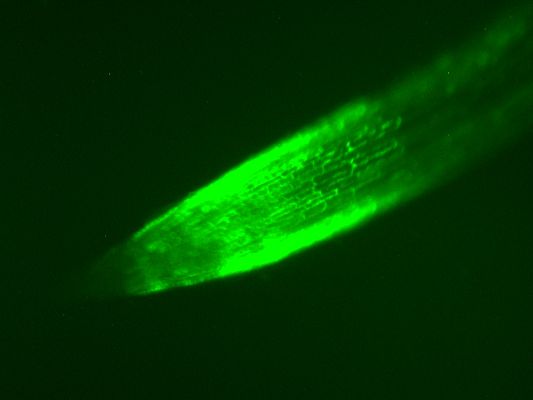 | ND | 28.8 ± 0.2 | 23.6 ± 0.6 | ND |
| AT1G61950.1 | Root hairs | 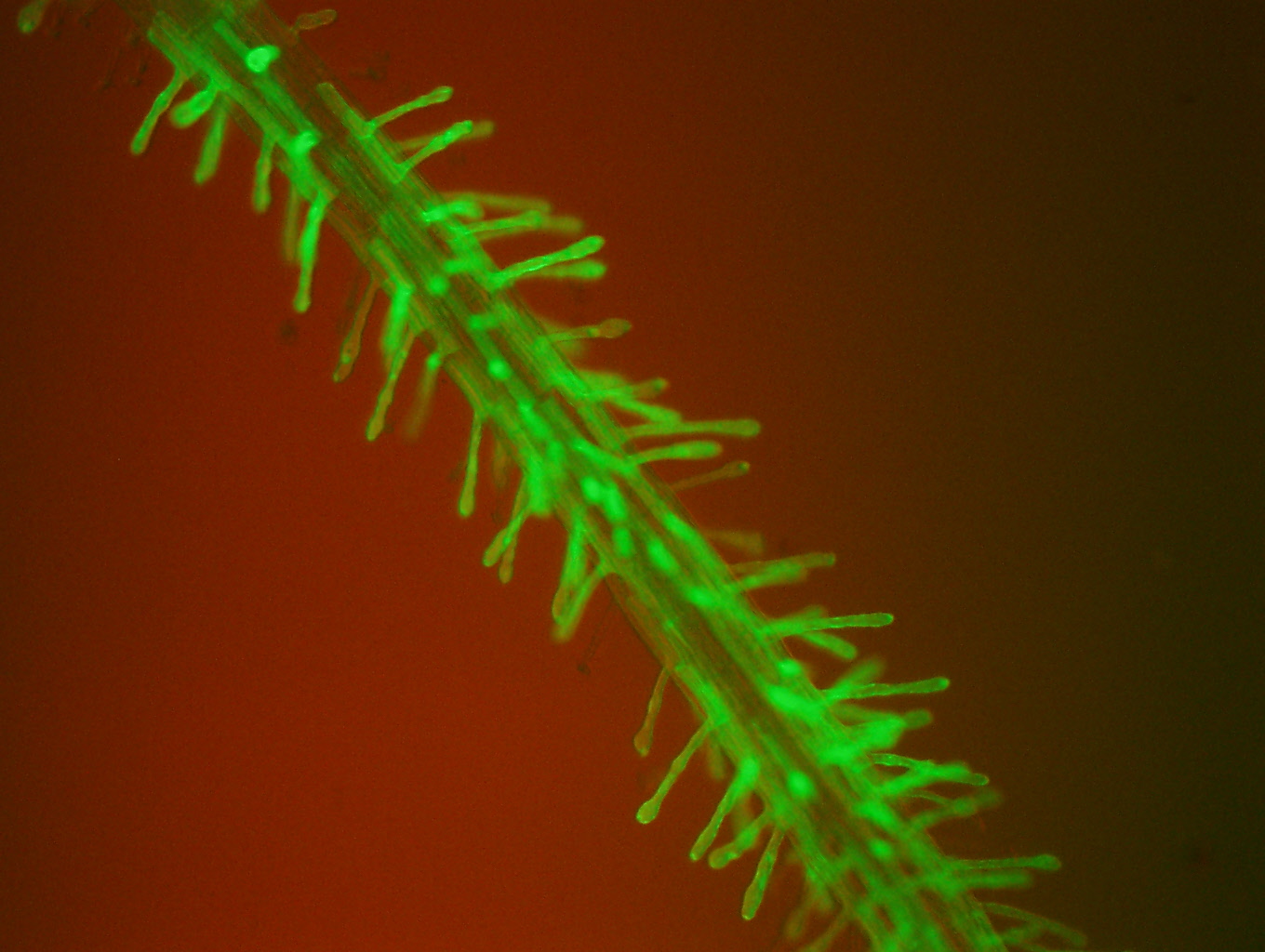 | ND | ND | 25.8 ± 0.2 | ND |
| AT1G69500.1 | Sepals, petals | 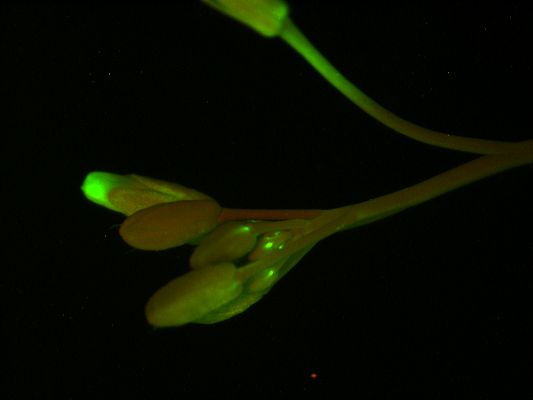 | 24.2 ± 0.4 | ND | ND | ND |
| AT2G02515.1 | Seeds | 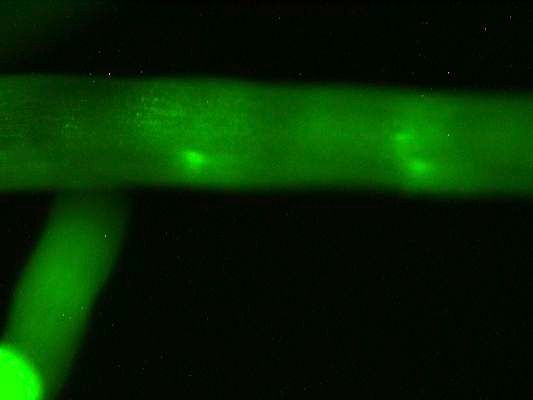 | ND | ND | 30.3 ± 0.3 | 21.3 ± 0.4 |
| AT2G17845.1 | Petals | 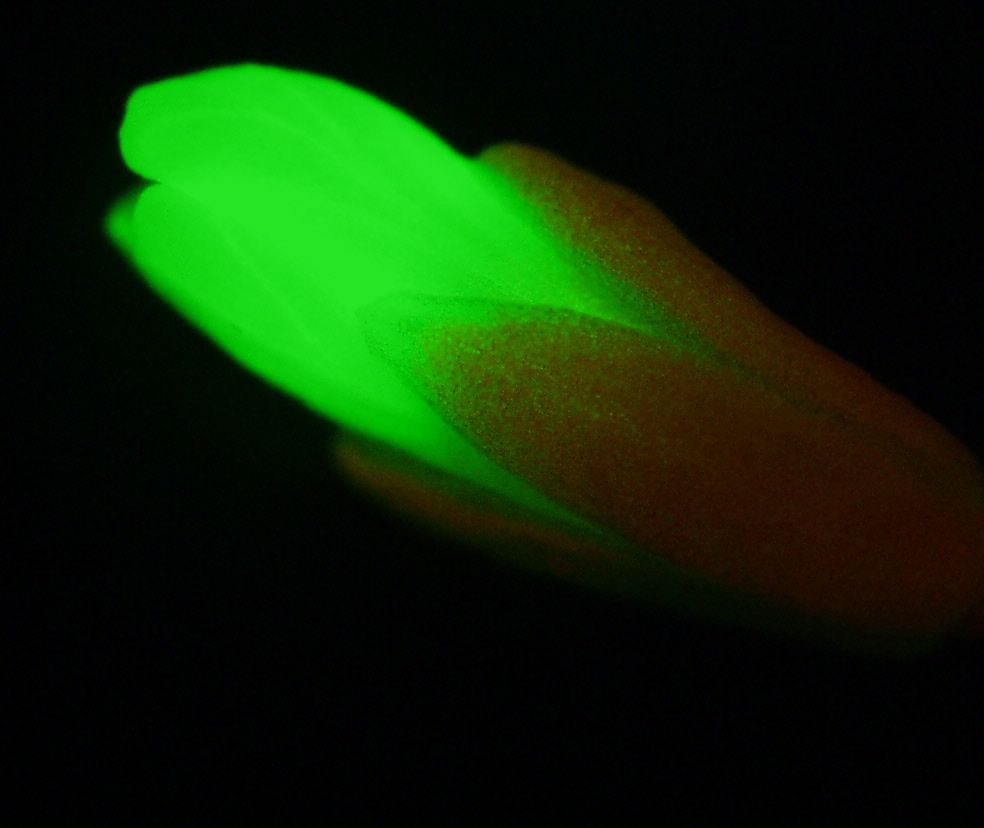 | 23.8 ± 0.5 | 34.5 ± 0.5 | 28.4 ± 1.0 | 30.1 ± 1.2 |
| AT2G24140.1 | Root vascular | 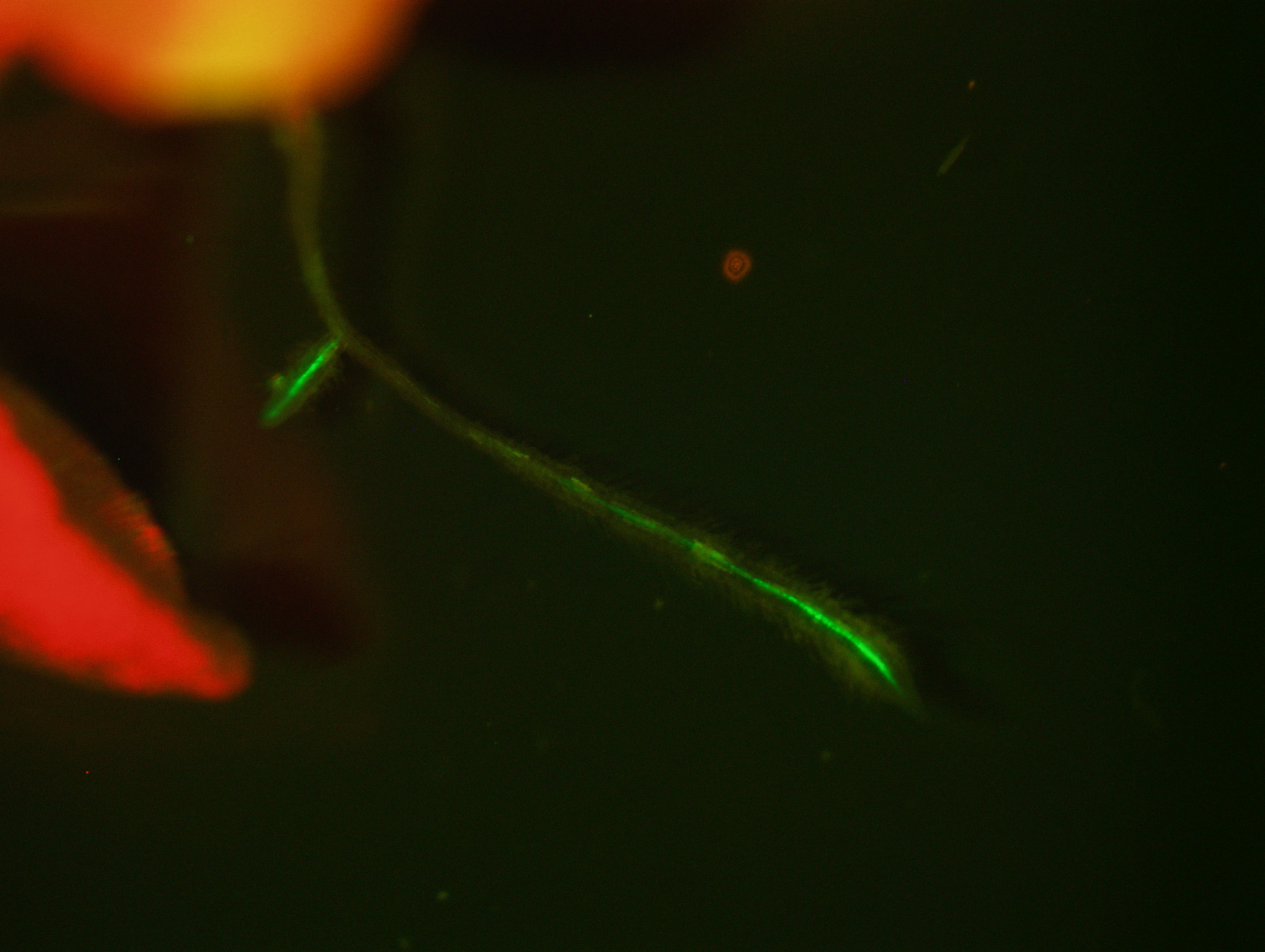 | 26.1 ± 0.3 | 28.9 ± 0.4 | 23.0 ± 0.4 | 27.0 ± 0.3 |
| AT2G40250.1 | Petals | 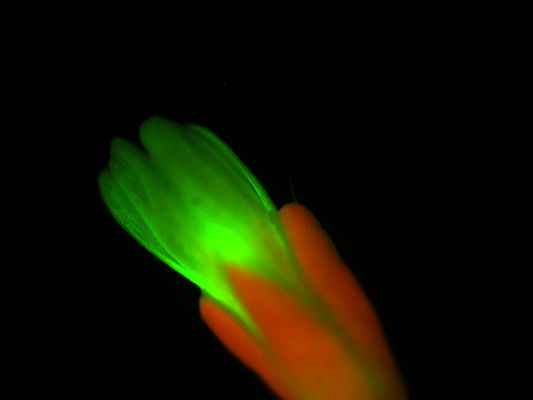 | 23.9 ± 0.1 | 34.0 ± 1.2 | 24.9 ± 0.3 | 27.3 ± 0.5 |
| AT3G58780.1 | Seeds | 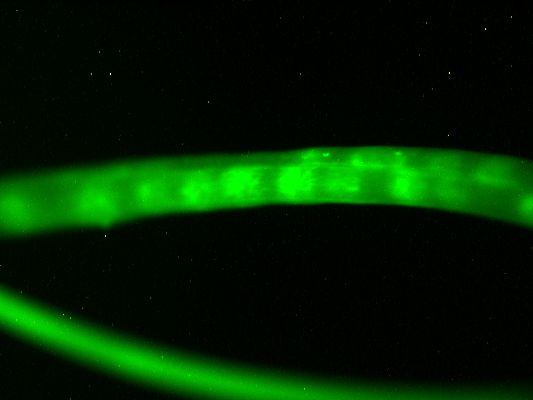 | 20.5 ± 0.2 | 29.9 ± 0.9 | 26.6 ± 0.1 | 19.7 ± 0.4 |

*Values shown are Ct ± SD from 3 biological replicates each with two technical replicates.

† ND = not robustly detected; Ct value > 35
